# Compacted Area with Effective Links (CAEL) for Data Dissemination in VANETs

**DOI:** 10.3390/s22093448

**Published:** 2022-04-30

**Authors:** Abdul Karim Kazi, Shariq Mahmood Khan, Waseemullah, Umer Farooq, Saman Hina

**Affiliations:** Department of Computer Science and Information Technology, NED University of Engineering and Technology, Karachi 75270, Pakistan; shariq@neduet.edu.pk (S.M.K.); waseemu@neduet.edu.pk (W.); umer@neduet.edu.pk (U.F.); samhaque@neduet.edu.pk (S.H.)

**Keywords:** ad hoc networks, reliability, link expiration, zone suppression, wireless communication

## Abstract

Vehicular ad hoc network (VANET) is a specialized form of wireless network that is solely intended for collaboration between vehicles. Several studies have shown that standard routing protocols cannot be implemented in VANETs because of their unique characteristics such as their significant count of vehicles on the network and the rapid evolution of the network’s design. Because VANET communication links are broken very frequently, it is necessary to address the routing consistency of these highly dynamic networks. The transmission of VANET data may result in a substantial amount of overhead in the routing process; thus, it is vital to address the issue of overhead to enhance the overall network performance. The proposed protocol named compacted area with effective links (CAEL) is designed to focus on decreasing overhead to achieve an enhancement in PDR performance inside the network. The communication between selected nodes that have been judged to be dependable in terms of geographical location and appropriate existing links between vehicles is focused on achieving this goal. With the inclusion of the reliability factor, it is possible to complete the important step of removing extraneous nodes, with the selection of the trustworthy nodes being made based on the link expiration time during the whole routing procedure. When compared to our previously published protocols, i.e., Dynamic Trilateral Enrollment (DyTE) and Reliable Group of Vehicles (RGoV), the results of the simulations demonstrate that CAEL has achieved an overall improvement in the performance of the network.

## 1. Introduction

Many people across the world are killed or injured every day due to traffic accidents [1]. These deaths and injuries could potentially be avoided if VANETs are used. Intelligent Transportation Systems (ITS) applications can benefit greatly from VANET’s support capabilities, which is why academics and researchers are interested in it [2,3,4,5]. VANETs are a cost effective means of providing communication amongst vehicles. Each vehicle on the road will be equipped with a wireless communication capability to facilitate the interconnection of wireless networks. A vehicle can receive or forward messages from nearby vehicles linked to the same network even if no supporting infrastructure is present. As a result, drivers will be able to keep track of changes in traffic conditions and other travel-related information as they are made accessible. VANETs have certain advantages in comparison to mobile ad hoc networks (MANETs), including enhanced computational power, increased transmission power, and a degree of projected mobility. The performance and quality of VANET generates major technical issues in existing systems which must be monitored to efficiently organize these types of connections. Frequent changes in network topology as well as the increased mobility of the network topology are likely to be the most difficult problems to solve [6]. When cars change their data rates or tracks in VANETs, the topology of the network also changes. Normally, the aforementioned modifications are not planned ahead of time and are entirely dependent on the road conditions and the actions of the drivers. VANET communication requires a routing mechanism that is both dependable and accurate in representing the topological needs of the network’s nodes, and development of such routing a mechanism is the major purpose of this research. Given that cars are driven at high speed on roads, disruptions in terms of data distribution facilities as a result of frequent interruptions in the connection are likely.

**Figure 1:**
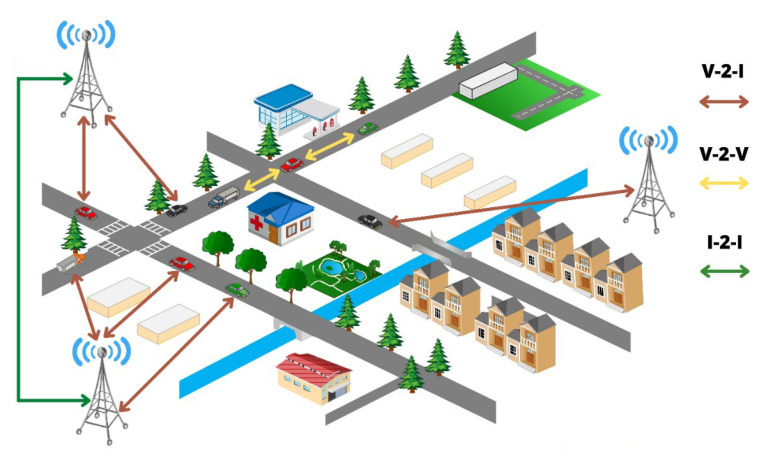
VANET’s architecture.

VANET’s architecture can be categorized as seen in Figure 1:

1. “Vehicle-to-Vehicle (V-2-V)”: The vehicles may directly communicate with other vehicles [7].
2. “Vehicle-to-Infrastructure (V-2-I)”: The local networks and cellular base towers make it possible for vehicles to communicate with one another at different geological locations.
3. “Infrastructure-to-Infrastructure (I-2-I)”: In addition to information being sent between many and distant sites, communication may take place among peer-to-peer infrastructures [8].

There is a cost associated with the implementation of vehicular communication systems. Primarily, hardware and software equipment, i.e., the vehicles must be equipped with an onboard unit (OBU) to support wireless communication and a sufficient amount of processing capability to process the information received from other nodes. VANET also require a proper capacity for storage, so that the data can be stored and processed before transmitting for wireless communication. The roadside units (RSU) must be upgraded to support intercommunication between vehicles on the road to enable the exchange of information related to road safety and infotainment.

VANET is different from traditional networks due to the absence of a centralized administrative authority that defines the rules for communication. This indicates that the node may act as a server and a client at the same time to share the information with other nodes. The following are some of the qualities of VANETs that make it more appealing:

1. Vehicles in VANET have a tremendous amount of power and storage.
2. Vehicles have an unlimited source of power, and their capacity to compute for the purposes of sensing and communicating is well supported.
3. Vehicle mobility can be predicted using velocity and coordinate information because of the presence of roadways.

The routing method for VANETs offers a substantial challenge because of the unique properties that must be addressed before these networks can be implemented. The data packets will be sent from the source to target node using the available intermediate vehicles. A high density of vehicles is not the only issue to address in the routing process, whereas crossing and traffic lights may generate a rupture in the network. In VANETs, routing protocol properties such as mobility restrictions and limited road patterns can be utilized using the Global Positioning System (GPS) coordinates and city maps to design an effective routing protocol.

The protocol proposed in the present study is an advancement of our previously published protocols, i.e., Reliable group of Vehicles(RGoV) [9] and Dynamic Trilateral Enrollment (DyTE) [10] through which the most reliable network pathways are taken into consideration while establishing a route.

The proposed protocol Compacted Area with Effective Links (CAEL) also uses the concept of minimizing the communication region as applied in DyTE and RGoV protocols. However, at the same time, CAEL calculates the expiration time for every connected link between the nodes to enhance the packet delivery rate (PDR) and reduce the routing overhead while keeping the latency time intact (if not minimized as compared to our previously published protocols). DyTE is based on minimizing the communication region of a node, only due to which the packet delivery performance increases; however, if the nodes get out of the minimized zone very quickly due to its high speed, then the performance of DyTE is affected—whereas RGoV first uses the suppression of the communication zone and then creates multiple clusters and each cluster contains a group of vehicles and selects only one cluster which is relatively closer to the destination vehicle. The shortcoming of RGoV lies in the selection process of a cluster group of vehicles that affects the network performance when it selects that cluster which is having few vehicles.

The remainder of this article is organized as follows: Section 2 contains a description of related work; Section 3 provides extensive information of the reliability model related to VANETs. The proposed routing method CAEL is discussed in Section 4; the performance of the proposed protocol CAEL is thoroughly evaluated as outlined in Section 5. Finally, Section 6 brings the paper to a conclusion.

## 2. Related Work

In VANET, the connection stability is a critical component to consider when creating a routing system. When developing routing algorithms, there are a variety of approaches that may be used to incorporate GPS coordinates and the route expiration time (RET) [11,12] to obtain the enhanced performance. An overview of the routing protocols is given where different techniques are applied to enhance the routing protocol’s efficiency. For the purpose of improving the quality of service (QoS), researchers devised a unique clustering-based technique [13] in which, instead of broadcasting the route request (RREQ) message to all cars, the algorithm is fitted with a clustering mechanism and the RREQ message is only communicated to the cluster head (CH). As a result, the CHs will be responsible for spreading routing information among the cluster participants. The route replay (RREP) message is transmitted to vehicles if a route is available; otherwise, the CH will send the RREQ message. It was projected that there would be less congestion and network overload as well as fewer connection failures because of this reduction in RREQ messages.

In order to establish the link with the most stable path, the authors of [14] proposed a routing protocol that integrates the direction and prediction of the path duration into the ad hoc on-demand distance vector (AODV) [15] protocol. The position of all vehicles is used to group them, and the route is chosen depending on the duration of the link. The authors did not account for the possibility of not having a sufficient number of nodes participating in a particular direction during the route finding process.

The authors [16] presented an improved QoS-based routing system that was dependent on the length of time a link has been in operation as well as the error rate experienced over the link. By synthesizing the network’s temporal, geographical, and environmental interactions, the article models and quantifies the robustness of links and routes, as well as their resilience to failure. In addition, a route selection method was provided on the basis of this information. The experiments conducted in this study showed that the ideal setting of the maximum routing hop count has not been taken into account throughout the routing process to date.

In [17], the authors proposed a QoS-based routing protocol that depends on the hop count, link duration, and connectivity, in order to deal with the dynamic topology and maintain the algorithm’s balance between stability and efficiency. However, the use of only global distance to represent the cumulative QoS of a routing path is insufficient.

The authors [18] proposed a routing protocol that makes use of software-defined network (SDN) and reinforcement learning to sense environmental data such as vehicle density and speed, and built the Q-learning-based cognitive routing algorithm to adopt alternative routing strategies depending on the environment.

The lifespan of a link was predicted using mobility and location data by the authors [19]. All node clocks are presumed to be synchronized using GPS. For instance, if two mobile nodes know their speed, direction, and position, then it is possible to predict the link’s lifespan. LET is calculated at each hop so that the time of the entire journey may be easily computed.

The authors in [20] proposed a new clustering technique to obtain a reliable low-latency multi-path routing scheme by incorporating the ant colony optimization (ACO) technique. However, even though the latency and RREQ messages are decreased, the technique does not determine the most stable path and does not take into account velocity fluctuations during direct engagement between the vehicles.

The authors of [10] devised a mechanism to focus on limiting the area of communication between the node. They created a trilateral zone which was is beneficial because it minimizes the participation of unnecessary nodes in the communication process. Since it only focuses on limiting the communication process, therefore, the nodes with high density can quickly go out of the trilateral zone and that drastically affects the performance of the network.

In another study, the authors [21] attained minimization in route disconnections. This was achieved because of the inclusion of a reliability factor and a route expiry period that takes hop counts into account. In order to discover the optimum communication routes, the most reliable paths between the source and the target are selected, whereas the authors in [22] achieved path reliability for data forwarding by identifying multiple roads at intersections.

A group of researchers [23] devised a protocol that determines the stability of a route based on the intensity of the signal being transmitted. In this protocol, the forwarding node adds the estimated intensity of the signal to the RREQ packets before sending them on to the next node. The only way to assess the overall signal strength of a route is to take the weakest signal strength of each of its individual connections. The response is sent back to the source node by the destination node, which chooses the path with the strongest predicted signal strength.

In [9], researchers have also reported that the following two techniques were combined to achieve the improved performance of the network: in the first technique, the area of communication is kept as small as possible in order to reduce unnecessary request traffic; in second technique, clusters of nodes were created based on their proximity to the centers of those clusters. At the end, whichever cluster was closest to the destination was selected for the communication. The problem arises when such a cluster is selected that has a very low number of nodes associated with it, and therefore the communication becomes disturbed when the node becomes unreachable. Furthermore, other important factors of a node such as speed and direction are neglected while making the cluster groups.

In an ad hoc network, a topology is used to create a temporary network and that topology is nothing more than a mix of mobile nodes and wireless networks [24]. In addition, it does not require any centralized management. The VANET is a cutting-edge technology that connects the automobiles of a new generation with the help of wireless networks [25]. It has the significance of research because of its potential to revolutionize the transportation system through the application of ITS [26]. With this technology, vehicles will be able to link to each other effectively [27] to make the transportation system more reliable. The creation of the VANET was motivated by the need to transmit information between cars on the road to avert accidents, hence increasing the overall safety of vehicles and drivers. Depending on the needs, all sensor data may be examined on the driver’s dashboard, sent to the RSU, or even broadcasted to nearby vehicles. Aside from providing road safety information, a wide variety of other uses for vehicular networks are listed, including gaming-related applications, traveling-related applications, multimedia-related applications, and access to the internet.

## 3. Reliability Model

The speed of vehicles on the road makes it difficult to maintain a reliable routing strategy in VANETs due to dynamic factors such as vehicle distribution and mobility patterns [28]. Therefore, to predict the duration of a steady route between vehicles, the vehicular characteristics must be determined which are discussed in the following sections.

### 3.1. Traffic Flow Foundation

There are two models used for traffic stream evaluation [29]. The first approach (known as the macroscopic approach) is based on a physical stream of traffic flow to describe traffic dynamics with the help of partial differential equations. The following correlations illustrate these aspects [30]:

(1) $$d_{m}=\frac{1000-(l_{m}\times p_{veh})}{p_{veh}}$$

(2) $$T_{m}=\frac{d_{m}}{v_{m}}$$

(3) $$q_{m}=\frac{1}{T_{m}}$$

where:

$p_{veh}$: Traffic density;
$d_{m}$: average distance between vehicles;
$v_{m}$: average velocity;
$T_{m}$: average time gap;
$q_{m}$: average traffic stream;
$l_{m}$: average vehicle length.

However, the other approach (microscopic approach) defines the vehicle behavior such as the change of lane, speeding up or down on the road. The connectivity of the network is assessed in the next step by analyzing the diversity of velocity instead of the vehicular flow of traffic. Vehicle speed is the most important factor in determining network topology dynamics. It also plays a significant role in determining the maximum communication time between two vehicles.

### 3.2. Reliability Framework

It may be summed up as the likelihood that two vehicles will be able to continuously communicate for a specified amount of time. The link between any two vehicles at given time has an availability interval of $T_{p}$; then, following relation is used to explain the term reliability:

(4) $$r\left(l\right)=P\{Availability\;until\;\left(t\;+\;T_{p}\right)\;|\;available\;at\;t\}$$

The calculations of the connection’s reliability requires the vehicles’ speed to be in standard distribution [31]. In this case, G(v) would be the probability distribution and g(v) would be the probability density function:

(5) $$g\left(v\right)=\frac{1}{\sigma \sqrt{2\pi }}e^{\frac{-{\left(v-\mu \right)}^{2}}{2\sigma^{2}}}$$

(6) $$G\left(v\leq V_{0}\right)=\frac{1}{\sigma \sqrt{2\pi }}\int_{0}^{V_{0}}e^{\frac{-{\left(v-\mu \right)}^{2}}{2\sigma^{2}}}dv$$

where μ and $\sigma_{2}$ depicts the average and variance of velocity, respectively, [32]. The relative velocity Δv and the time duration *T* is utilized to calculate the distance. Because the two arbitrary variables $v_{1}$ and $v_{2}$ are normally distributed, the difference between those two will be normally distributed as well. The greatest distance upon which two vehicles may still communicate is 2H, while each vehicle’s communication range is given as *H*. The following equation may be used to compute the probability density function for the communication duration *T*:

(7) $$f\left(T\right)=\frac{4H}{\sigma_{\Delta v}\sqrt{2\pi }\times T^{2}}e^{\frac{-{\left(\frac{2H}{T}-\mu_{\Delta v}\right)}^{2}}{2\sigma_{\Delta v}^{2}}}forT\geq 0$$

where $\mu_{\Delta v}$ and $\sigma_{\Delta v}^{2}$ depict the average and variance of relative velocity, respectively. Each vehicle should be fitted with a GPS device that can provide the speed, direction, and position information. Equation (8) provides a mathematical representation of the specific link’s continuity between two vehicles:

(8) $$T_{p}=\frac{H-L_{ij}}{v_{ij}}=\frac{H-Euclidean_{dist}}{|v_{i}-v_{j}|}$$

Euclidean distance formula is used to calculate the distance between vehicle *i* and *j*. The function f(T) can be integrated from time *t* to $t+T_{p}$ to calculate the possibility of a connection, reachable at *t* for a duration of $T_{p}$. The following Equation (9) can be used to determine the reliability of a link at a specific point in time:

(9) $$r_{t}\left(l\right)=\left\{\begin{array}{cc} \int_{t}^{t+T_{p}}f\left(T\right)dt & \mathrm{if}\;T_{p}>0\\ 0 & \mathrm{otherwise} \end{array}\right.$$

The Gauss error function can be used to derive the integral in Equation (9) [33],

(10) $$r_{t}\left(l\right)=Erf\frac{\left(\frac{2H}{t}-\mu_{\Delta v}\right)}{\sigma_{\Delta v}\sqrt{2}}-Erf\frac{\left(\frac{2H}{t+T_{p}}-\mu_{\Delta v}\right)}{\sigma_{\Delta v}\sqrt{2}}\;when\;T_{p}>0$$

where *Erf* stands for;

(11) $$Erf\left(T\right)=\frac{2}{\sqrt{\pi }}\int_{0}^{T}e^{-t^{2}}dt,-\infty <T<+\infty$$

### 3.3. Route Reliability

Multiple possible routes exist between the origin and the destination node in VANETs, whereas all routes are a set of connected links. The number of connections that have been formed is shown as $\delta :l_{1}=\left(s,n_{1}\right),l_{2}=\left(n_{1},n_{2}\right),\ldots ,l_{\delta }=\left(n_{\delta },d\right)$ on any particular route by keeping the generality intact. Every single link $l_{c}\left(c=1,2,\ldots ,\;\delta \right)$ is represented by $r_{t}\left(l_{c}\right)$, whereas Equation (12) interprets the reliability of a link for a particular route X.

(12) $$R\left(X\left(s,d\right)\right)=\prod_{c=1}^{\delta }r_{t}\left(l_{c}\right),\;where\;l_{c}\in X\left(s,d\right)$$

The multiple reliability product over the existing links for any route is used to classify the route’s reliability. Assume that there are Ω numbers of routes between the source and the target node. The source node will choose the best possible route, i.e., $Q\left(s,d\right)=X_{1},X_{2},\ldots ,X_{\Omega }$ amongst them, using the relation represented in Equation (13).

(13) $$arg\;max_{X\in Q(s,d)}R\left(X\right)$$

## 4. Proposed Protocol

The proposed protocol CAEL is based on two mechanisms that work together, i.e., the suppression of the communication region and expiration details of the links to improve the performance. Therefore, the information about the process of creating a trilateral region and the mechanism is mentioned for the identification of any node whether it lies inside or outside the said zone is discussed and then the mechanism of RF, RET and LET is also explained.

### 4.1. Trilateral Region’s Construction

**Figure 2:**
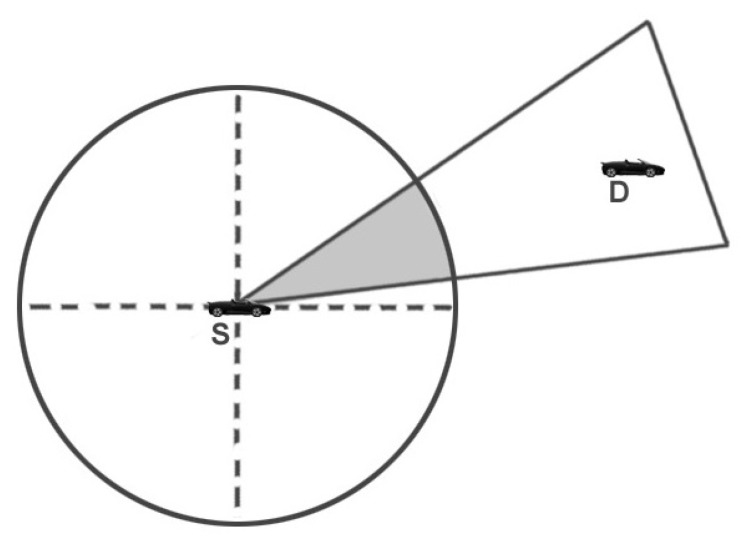
Clipped communication zone.

**Figure 3:**
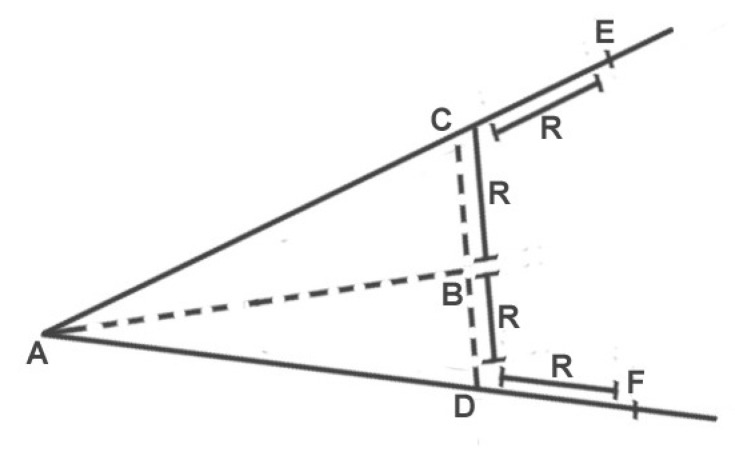
Trilateral zone.

As mentioned in Figure 2, the communication region of node *S* is circular in shape, as the goal is to construct a trilateral region (highlighted as grey colored) so that only a limited number of reliable nodes may participate in the communication process. It is presumed that vehicles have an on-board navigation system. Each vehicle is considered to have GPS receivers and preloaded street maps. GPS receivers identify the location and direction, which is helpful for nodes in calculating vehicle density. The trilateral zone, ▵AEF, must be constructed to limit the communication area (as illustrated in Figure 3). The last known coordinate information of the target node is utilized by the source node to compute the slope and the distance between them using Equations (14) and (15), respectively.

(14) $$m_{straight}=\frac{\Delta y}{\Delta x}=\frac{y_{D}-y_{S}}{x_{D}-x_{S}}$$

(15) $$Euclidean_{dist}=\sqrt{{\left(\Delta y\right)}^{2}+{\left(\Delta x\right)}^{2}}$$

Then, we can determine the coordinate information of the perpendicular points *C* and *D* by solving Equation (15) with Equation (16).

(16) $$m_{perpendicular}=\left(\frac{1}{-m_{straight}}\right)$$

After calculating the perpendicular slope, we must use the Euclidean distance formula to obtain the distance between $\bar{BC}$ and $\bar{BD}$. We may also obtain the coordinates of *E* by finding the slope of straight-line $\bar{AC}$ because that straight-line connects all three points; therefore, lines $\bar{AC}$ and $\bar{CE}$ have identical slopes. Similarly, we can calculate point *A*, *D*, and *F* as they are located on a straight-line by using the slope of line $\bar{AD}$.

### 4.2. Members Identification Mechanism in Trilateral Region

**Figure 4:**
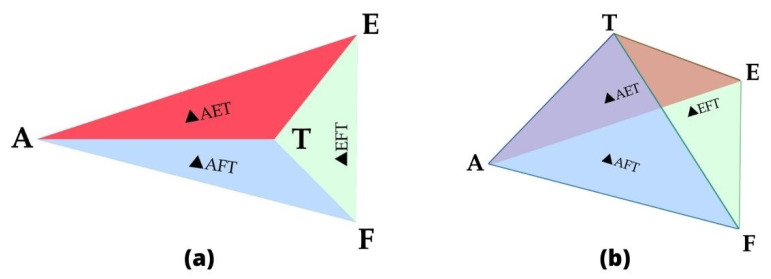
Random node T (**a**) lies inside the region; and (**b**) lies outside the region.

The receiving node must be in the trilateral zone of the transmitting node to participate in the routing procedure. The sender node adds the list of neighbors that lies within its calculated trilateral region by computing the area of the triangle, ▵AEF, as illustrated in Figure 4. The area of ▵AEF is calculated using the Equation (17).

(17) $$Area\left(▵AEF\right)=|\frac{y_{E}\left(x_{A}-x_{F}\right)-y_{F}\left(x_{A}-x_{E}\right)-y_{A}\left(x_{E}-x_{F}\right)}{2}|$$

There are two possibilities shown in Figure 4 about the position of a random node *T* which lies inside and outside, respectively. The node *T* will be allowed to participate in the communication process if it lies inside the trilateral region or else it will be dropped. Equation (18) is used to determine whether a node is located inside or outside the trilateral region.

(18) Area(▵AEF)=Area(▵AET+▵AFT+▵EFT)

### 4.3. Link Expiration

At this stage, the proposed protocol CAEL uses the GPS coordinates of the source and the destination node through which a limited area (in a triangular shape) is calculated, after this calculation, all the neighbor nodes will be identified inside the (limited) region. As a result, the list of neighbor nodes is created and link expiration time (LET) of each node in the list is also calculated. After this, the average of all nodes LET is calculated. Then, the list of nodes is further shrunken by comparing each LET of the node by the average LET (which has already been calculated). If the node’s LET is less than the average LET of the list, then that node is subtracted and only those nodes will be placed intact in the list whose LET is greater than the average of all LET nodes.

(19) $$if(LET_{node}-LET_{avg.})\geq 0$$

The proposed protocol is different from our previously published protocols (DyTE) because it does not only rely on a limited zone but also does not use a clustering technique as RGoV. The link expiration in CAEL helps in the identification of the time duration for which the links would be available. The proposed protocol CAEL makes use of the node’s speed and direction to calculate the effective routes for the communications.

To understand how the reliability factor is calculated and which parameters are required for it, the details are as follows; the reliability factor (RF) is a technique to determine the most reliable route which considers the “Route Expiration Time (RET)” and “Hop Count (HC)” while choosing a routing path. The selection of a reliable route is based on the RF value; and a greater value of RF indicates higher route reliability than is appropriate for the transfer of data packets.

#### 4.3.1. RF

When it comes to transmitting data from source to destination, RF selects the route with the shortest RET and the least count of hops to obtain the most reliable path [34]. RF is essentially a discrepancy between the normalized values of RET and HC which can be computed using Equation (20).

(20) $$RF=\frac{RET}{RET_{max}}-\frac{HC}{HC_{max}}$$

The calculation of RF requires the estimations of multiple necessary parameters such as RET, HC, $RET_{max}$ and $HC_{max}$; these important parameters and the method to calculate the RF are subsequently discussed.

#### 4.3.2. RET

**Figure 5:**
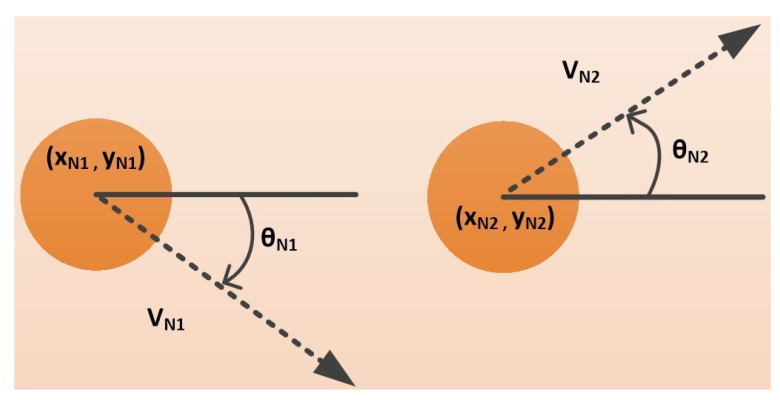
Parameters used for calculating Link Expiration Time (LET) between two nodes.

RET specifies the minimum duration amongst all connections between the source and target node, whereas LET specifies the estimated duration for which the connection between any two nodes stays active [35]. A route with a higher RET is a considered a more dependable route. Figure 5 illustrates two nodes [36], i.e., where node $N_{1}$ has $\left(x_{N1},y_{N1}\right)$ coordinates along with the velocity $v_{N1}$ which moves at an angle $\theta_{N1}$ whereas node N2 have $\left(x_{N2},y_{N2}\right)$ coordinates along with the velocity $v_{N2}$ which moves at an angle $\theta_{N2}$. Furthermore, both nodes have same the communication range *R*. Then, the LET between two nodes can be calculated using Equation (21).

(21) $$LET=\frac{-\left(\left(\alpha \times \beta \right)+\left(\gamma \times \lambda \right)\right)+\sqrt{\left(\alpha^{2}+\gamma^{2}\right)\times R^{2}-{\left(\left(\alpha \times \lambda \right)+\left(\gamma \times \beta \right)\right)}^{2}}}{\alpha^{2}+\gamma^{2}}$$

where,

$\alpha =v_{N1}\times cos\theta_{N1}-v_{N2}\times cos\theta_{N2}$
$\beta =x_{N1}-x_{N2}$
$\gamma =v_{N1}\times sin\theta_{N1}-v_{N2}\times sin\theta_{N2}$
$\lambda =y_{N1}-y_{N2}$

The *RET* is actually the lowest *LET* of all possible network links, determined using Equation (22).

(22) $$RET=min(LET_{1},LET_{2},LET_{3},\ldots ,LET_{n})$$

#### 4.3.3. $RET_{max}$

**Figure 6:**
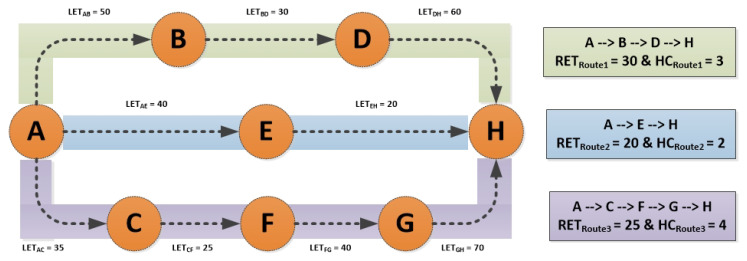
Route Expiration Time (RET) and Hop Count (HC) for all possible routes.

$RET_{max}$ is the highest value of RET across all possible routes at the destination (which can be calculated using Equation (23)). As explained earlier, the LET between any two nodes can be acquired using Equation (21) and the minimum LET in the possible route tagged as the RET of that route. As depicted in Figure 6, multiple possible routes are available if node A aims to send data to node *H*. The connection between any two nodes signifies the LET of both nodes, for example, the LET between node *A* and node *B* is equal to 50.

(23) $$RET_{max}=max\left(RET_{Route1},RET_{Route2},\ldots ,RET_{RouteN}\right)$$

Since $RET_{Route1}=30,RET_{Route2}=20\;\&\;RET_{Route3}=25$

Therefore

$RET_{max}=max\left(RET_{Route1},RET_{Route2},RET_{Route3}\right)$

$RET_{max}=max\left(30,20,25\right)$

$RET_{max}=30$

**Figure 7:**
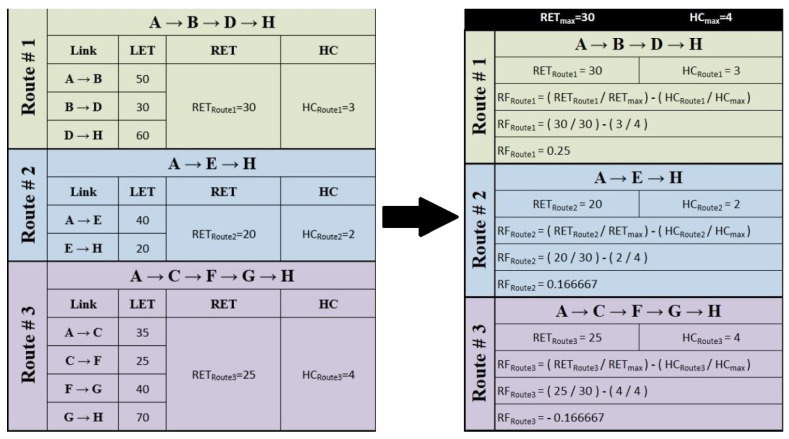
Tabular explanation and calculation of the best possible Reliability Factor (RF).

Figure 7 summarizes Figure 6 for a better understanding of $RET_{max}$.

#### 4.3.4. $HC_{max}$

HC reflects the total number of hops it takes to go from one to another whereas $HC_{max}$ specifies the highest number count of hops from all possible routes between the source node and the target node (as mentioned in Figure 6):

(24) $$HC_{max}=max\left(HC_{Route1},HC_{Route2},\ldots ,HC_{RouteN}\right)$$

Since $HC_{Route1}=3,HC_{Route2}=2\;\&\;HC_{Route3}=4$

Therefore

$HC_{max}=max\left(HC_{Route1},HC_{Route2},HC_{Route3}\right)$

$HC_{max}=max\left(3,2,4\right)$

$HC_{max}=4$

### 4.4. Route Discovery

The proposed protocol (CAEL) initiates the route discovery phase when node A sends an RREQ packet to all nearby nodes to send data packets to node H but does not have routing information for that specific node inside the trilateral zone. The node analyzes its routing table when it receives an RREQ packet to identify the reverse route to the source node. If a route to the source node already exists in the routing table, it is updated; otherwise, a reverse route is created. To find the shortest route to reach the target node, the receiving node first calculates LETs between RREQ sending nodes and its current node, and then selects the shortest one between the current node and its source node. After that, it increases the value of the hop count and transmits the RREQ packet to neighbor nodes. Intermediary nodes may receive several copies of the same RREQ message from different surrounding nodes, but it just drops the request. Intermediary nodes produce RREP messages if any of them have an active route to the target node, and it selects the most reliable route if any of the intermediate nodes are targets themselves.

### 4.5. Route Selection

When the very first RREQ packet is received at the target node H within the trilateral zone, a timer is started that waits for a specified amount of time. During this time, the target node begins the collection of all other RREQ packets that attempt to reach it. When the timeout event occurs, the target node computes the RF value for each path gathered from the source to the target and chooses the routing path with the highest value of RF.

To understand how CAEL’s route selection process works, consider the network design shown in Figure 6 where multiple paths are available to reach from the source to the target node. The calculation of the best possible RF is shown in Figure 7 and it can be observed that $RF_{Route1}$ has the highest calculated RF value among all other viable routes. Therefore, Route#1 will be selected for the data transfer.

## 5. Settings for Performance Assessment

This section examines the effect on the performance of RGoV [9], DyTE [10], COOP [37], NCA(MPR) [38], CACA [39], and CAEL at various network densities. The network density has been demonstrated by deploying up to 300 nodes over an area of 2500 m × 2000 m. Every individual node in the network may travel at a maximum speed of 50 km per hour. Between random source–destination connections, a maximum of 100 connections are established with the packet 512 bytes in size.

The IEEE 802.11p standard is widely used for wireless connectivity in vehicular communication. IEEE 802.11p is considered the cross-layer standard for Physical Layer/Medium Access Control (PHY/MAC), incorporating orthogonal frequency-division multiple access (OFDMA) and a frequency spectrum specified for vehicular communication of 5.9 GHz. Carrier Sense Multiple Access with Collision Avoidance (CSMA/CA) is the foundation of the IEEE 802.11p MAC. A node initially senses the channel before transmission, and during the transmission process, the node and channel become inactive for a short period of time. The transmission only resumes when the channel is idle. If the wireless channel is busy, an arbitrary back-off mechanism is implemented with a contention window (CW) and only starts to transmit at the lapse of the back-off timer.

The performance of the proposed protocol is evaluated and compared with existing routing protocols using the NS-2 simulator, because it is an open source simulator designed specifically for research in computer communication networks based on discrete events [40] and object-oriented simulations. Since its inception, NS-2 has piqued the interest of industry, academia, and the government. It has been under constant investigation and enhancement for years. In fact, the NS-2 simulator was used in recent research [39,41,42,43,44,45,46]. NS-2 contains modules for numerous network components, such as routing, transport layer protocols (TCP and UDP), and applications; therefore, the researchers can simply use the scripting language to configure a network and observe the results generated by NS-2 to investigate network performance.

A realistic urban scenario is designed using the tool called the Simulation of Urban Mobility (SUMO) [47]. SUMO also delivers the most frequently used information regarding the traffic conditions via the use of road directions, edges information, and vehicle speed. Additionally, SUMO generates the mobility trace file that defines the wireless mobile network, which consists of various randomly distributed nodes that follow the road’s behavior. Furthermore, a piece of map is extracted using the OpenStreetMap (OSM) [48] containing a portion of the city Karachi (Pakistan), which is converted using SUMO [49] to implement the vehicular traffic scenario.

In each result (discussed in Section 5.1.1, Section 5.1.2 and Section 5.1.3), the x axis represents the variation in node density whereas the y axis represents the performance metric. The summary of the simulation settings and its parameters is given in Table 1.

### 5.1. Performance Metrics

The following metrics are used to evaluate the proposed routing protocol’s performance:

1. Packet delivery ratio (PDR): The ratio of packets that are transmitted from the source to the destination.
2. Routing overhead: This is defined as the additional number of routing packets sent across all nodes to reach the destination. A lower count of overhead packets indicates better performance.
3. Average end-to-end (E2E) delay: This indicates the average time interval between packet transmission and reception.

#### 5.1.1. PDR

**Figure 8:**
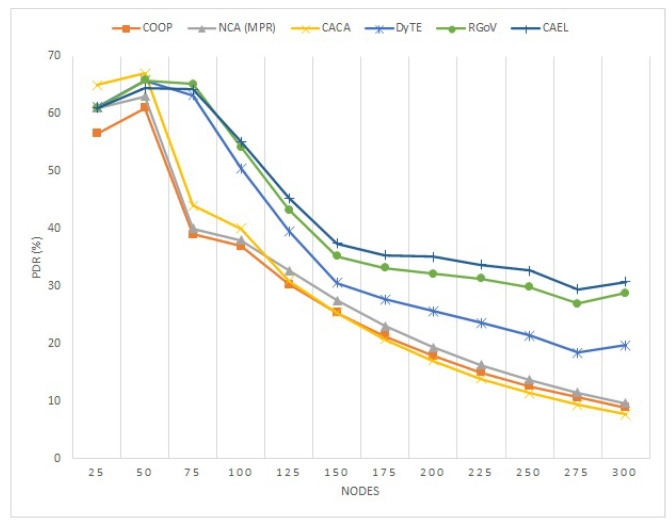
Packet delivery ratio against various number of nodes.

The impact of network density on the PDR performance of the protocols COOP, NCA(MPR), CACA, DyTE, RGoV, and CAEL is depicted in Figure 8. Due to the nature of wireless communication where no centralized administration is present, the higher the number of nodes, then the higher the number of route requests which is generated to reach the destination, causing network congestion. It can be observed in Figure 8 that when the number of nodes is low in density, the PDR achieved by every protocol is generally higher, and when the number of nodes starts getting higher, the PDR is drastically decreased. On the other hand, the proposed protocol CAEL delivers a 3.5% and 17% improved packet delivery ratio than RGoV and DyTE, respectively, and its performance becomes improved and stable, especially in dense networks. Because other protocols such as DyTE suppress the communication zone (only) and protocols such as COOP, NCA(MPR), CACA, and RGoV work on the principle of clusters, CAEL chooses the most dependable routes to minimize connection failures. While DyTE and RGoV both choose the shortest route between sources and destinations without regard for node speed or direction, the proposed protocol (CAEL) chooses the most reliable and efficient routing path by taking the node’s speed and direction into account and eventually increasing the data packet delivery. In addition, CAEL also considers the important factor of link expiration time while finding effective routes.

#### 5.1.2. Overhead

**Figure 9:**
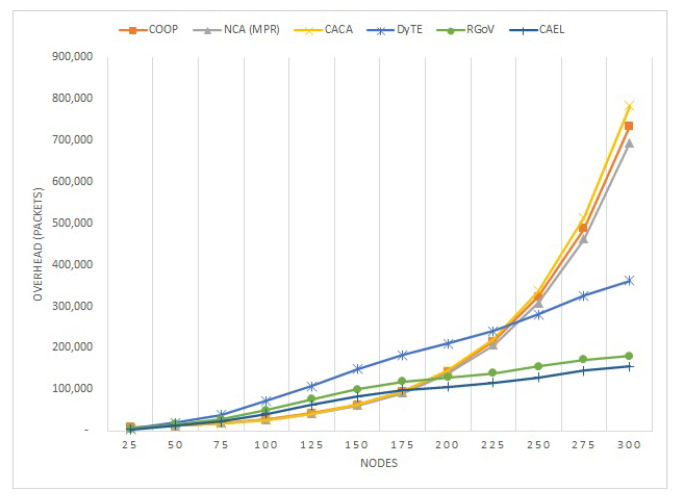
Routing overhead against various number of nodes.

The routing overhead of COOP, NCA(MPR), CACA, DyTE, RGoV, and CAEL is presented against different network densities in Figure 9 with nodes deployed in a 2500 m × 2000 m topological region. As the number of nodes rose, so did the routing burden. As seen in Figure 9, the CAEL’s network routing load is 16% less than that of RGoV and 50% less than the rest of the other mentioned protocols, especially in dense networks, because CAEL’s adoption of a dependable route resulted in a reduction in route disconnections. The reduced chances of route failures result in a decrease in the commencement of route rediscovery and maintenance procedures, and therefore CAEL’s reliable approach has a lower network routing burden than other aforementioned protocols.

#### 5.1.3. E2E

**Figure 10:**
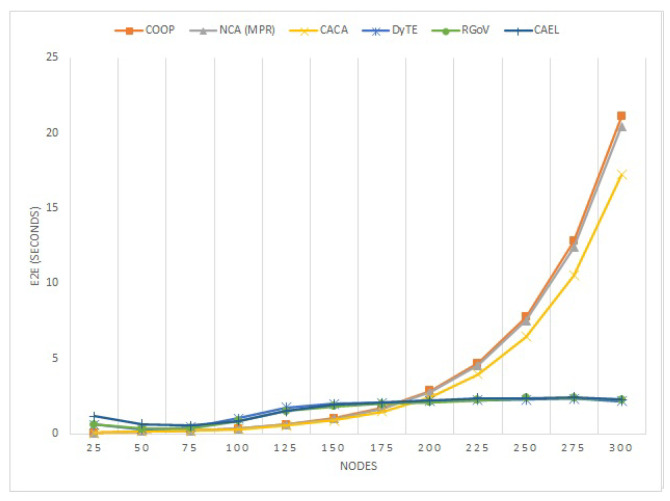
End-to-end delay against various number of nodes.

The average E2E latency is depicted in Figure 10 for various node counts. Since the proposed protocol is based on the compaction of the communication region, when the node density is low, the number of nodes participating therefore also becomes very limited and even no node participates in the communication process, which leads to an increase the latency time. It can be seen that with the rise in nodes count, the E2E latency for all protocols increased but due to the dependable route selection, the CAEL took roughly the same amount of time on average to transport the data packet as the RGoV and DyTE. In contrast, CAEL easily outperformed the remaining COOP, NCA(MPR), and CACA protocol because CAEL works on the principle of minimizing the communication region of a node and also selects the best route based on the availability of the link before its expiration.

COOP, NCA (MPR), and CACA are solely based on improving the MPR selection to reduce the network overhead, whereas DyTE and RGoV control overhead packets using the time and distance information of the nodes, and RGoV also adds a clustering technique on top of that to improve the performance by adding a reliability factor. The proposed protocol (CAEL), on the other hand, not only minimizes the communication region but also checks the link expiration of each connected route to determine the best possible way to reach the destination. The results show that the overall performance of CAEL is far more effective than that of COOP, NCA (MPR), CACA, DyTE, and RGoV.

It is observed from the results that the performance of the proposed protocol is superior to the other mentioned protocols, but in some scenarios, the performance of CAEL may deteriorate and affect the computation performance. Those multiple scenarios are when very few nodes are identified in the trilateral region and those nodes appear to reside on the border of the trilateral region, which means that they can leave of the trilateral region very quickly. Secondly, when the calculated LETs between all nodes are very low, and third, due to the large number of HCs, since RF is based on RET and HC, if HC is very high, the reliability would be affected.

## 6. Conclusions

In this study, a novel routing system called the Compacted Area with Effective Links (CAEL) is proposed. This routing protocol first compacts the communication area and then determines the most reliable route inside the compacted region based on the RF. RF takes into account RET and HC to identify the most reliable routing path with the fewest hops. The value of RF is used to determine the most reliable path for data transfer; a higher value of RF implies a more reliable method of data transmission. Upon further research and the analysis of the simulation results, it was discovered that DyTE and RGoV impose a large amount of routing strain on the network as a result of the blind flooding of RREQ packets that occurs during the route discovery stage. This flooding process increases the number of times the RREQ packet is re-transmitted throughout the network, resulting in increased network congestion and significant network performance degradation. According to the reported literature, this re-transmission of RREQ packets is known as a ‘Broadcast Storm’. To re-establish the broken path, it was necessary to undertake route maintenance or route rediscovery method which resulted in higher network traffic and a detrimental impact on network performance.

We developed a routing method that is based on the dependability factor, which may select a highly stable route, hence boosting the longevity and performance, especially for high-density networks. In the future, we aimed to boost throughput and PDR while significantly reducing the latency time. This may be accomplished by tweaking the parameters of RET as well as by adding conditions to handle those nodes that reside near the border of the trilateral region to avoid link disconnections.

## Figures and Tables

**Table 1:** Simulation Settings.

Parameter	Settings
Mobility	Manhattan
MAC Protocol	IEEE 802.11p
Network Simulator	NS-2
Channel Type	Wireless
Propagation Model	Two Ray Ground
Antenna	Omni-Antenna
Transmission Range	250 m
Traffic Type	CBR
Protocol Type	UDP
Max. Speed	50 Km/h
Buffer Length	50 packets
Number of Vehicles	25, 50, 75, 100, 125, 150, 175, 200, 225, 250, 275, 300
Number of Simulations	15
Simulation Area	2500 m × 2000 m
Simulation Time	300 s
Routing Protocols	COOP, NCA(MPR), CACA, DyTE, RGoV, and CAEL

## Data Availability

Not Applicable.

## Abbreviations

The following abbreviations are used in this manuscript:

ACO	Ant Colony Optimization
CAEL	Compacted Area with Effective Links
CH	Cluster Head
DyTE	Dynamic Trilateral Enrollment
HC	Hop Count
E2E	End-to-End
GPS	Global Positioning System
ITS	Intelligent Transportation Systems
I-2-I	Infrastructure-to-Infrastructure
LET	Link Expiration Time
MANET	Mobile Ad Hoc Network
NS-2	Network Simulator 2
OBU	Onboard Unit
OSM	OpenStreetMap
PDR	Packet Delivery Ratio
QoS	Quality of Service
RGoV	Reliable Group of Vehicles
RSU	Roadside Unit
RF	Reliability Factor
RET	Route Expiration Time
RREQ	Route Request
RREP	Route Reply
SDN	Software-Defined Network
SUMO	Simulation of Urban Mobility
VANET	Vehicular Ad Hoc Network
V-2-V	Vehicle-to-Vehicle
V-2-I	Vehicle-to-Infrastructure
